# Supplementary material for: A Novel Aldisine Derivative Exhibits Potential Antitumor Effects by Targeting JAK/STAT3 Signaling
Source: Mar Drugs. 2023 Mar 29;21(4):218. doi: 10.3390/md21040218 (PMC10141377; doi:10.3390/md21040218)
Supplement: Supplementary file 1 [file marinedrugs-21-00218-s001.zip › marinedrugs-2276570-supplementary.pdf]

A novel aldisine derivative exhibits potential antitumor effects by targeting JAK/STAT3 signaling

1. General procedure for Compounds **8a~8c**, **9a~9c**.
2. General procedure for Compounds **10a~10c**, **11a~11c**.
3. Tabel S1: The high-throughput-screen result of **aldisine** derivatives.
4. Figure S1: The high-throughput screen of compounds graph.
5. The data of ^{1}H and ^{13}C NMR of all the compounds.

1. General procedure for Compounds **8a~8c**, **9a~9c**.

KOH (33.0 mmol) was added to a solution of 7 (3.3 mmol) in DMSO (20 mL) at 25 °C for 1 h; then 1, ω-dibromoalkan (49.5 mmol) was added and the mixture was stirred for 0.5 h. Then water (100 mL) was added, and the mixture was extracted with EtOAc (3 × 100 mL). The combined organic layers were dried with $MgSO_4$ and concentrated under a vacuum. The resulting residue was purified by chromatography on silica gel to give the compounds **8a~8c**, **9a~9c**.

2,3-dibromo-1-(4-bromopropyl)-6,7-dihydropyrrolo[2,3-c] azepin-8(1*H*)-one (**8a**)
White solid (607 mg, yield 42%); ^{1}H NMR (600 MHz, Chloroform-*d*) δ 6.68 (d, *J* = 9.9 Hz, 1H), 6.22–6.15 (m, 1H), 6.05 (dt, *J* = 9.8, 6.4 Hz, 1H), 4.52 (t, *J* = 7.2 Hz, 2H), 3.54 (t, *J* = 6.2 Hz, 2H), 3.41 (t, *J* = 6.6 Hz, 2H), 1.98–1.86 (m, 4H); ^{13}C NMR (150 MHz, Chloroform-*d*) δ 162.68, 126.62, 126.55, 126.23, 125.22, 112.88, 99.82, 47.66, 38.84, 32.94, 29.80, 29.65; HRMS calcd for $C_{12}H_{13}Br_3N_2O$ $[M + H]^+$ 441.8558, found 441.8551.

2,3-dibromo-1-(5-bromobutyl)-6,7-dihydropyrrolo[2,3-c] azepin-8(1*H*)-one (**8b**)
White solid (596 mg, yield 40%); ^{1}H NMR (600 MHz, Chloroform-*d*) δ 6.72 (d, *J* = 9.9 Hz, 1H), 6.14 (t, *J* = 6.1 Hz, 1H), 6.08 (dt, *J* = 9.9, 6.3 Hz, 1H), 4.52 (t, *J* = 7.6 Hz, 2H), 3.57 (t, *J* = 6.1 Hz, 2H), 3.44 (t, *J* = 6.6 Hz, 2H), 1.93 (p, *J* = 6.9 Hz, 2H), 1.83 (q, *J* = 7.8 Hz, 2H), 1.52 (p, *J* = 7.6, 7.2 Hz, 2H); ^{13}C NMR (150 MHz, Chloroform-*d*) δ 162.62, 126.59, 126.37, 126.05, 125.17, 112.80, 99.64, 48.29, 38.78, 33.54, 32.09, 30.01, 25.11; HRMS calcd for $C_{13}H_{15}Br_3N_2O$ $[M + H]^+$ 455.8714, found 453.8717.

2,3-dibromo-1-(6-bromobutyl)-6,7-dihydropyrrolo[2,3-c] azepin-8(1*H*)-one (**8c**)
White solid (6000 mg, yield 39%); ^{1}H NMR (600 MHz, Chloroform-*d*) δ 6.68 (d, *J* = 9.9 Hz, 1H), 6.20 (t, *J* = 6.1 Hz, 1H), 6.04 (ddd, *J* = 10.1, 7.0, 6.0 Hz, 1H), 4.49–4.45 (m, 2H), 3.52 (t, *J* = 6.2 Hz, 2H), 3.38 (td, *J* = 6.8, 0.9 Hz, 2H), 1.85 (p, *J* = 7.0 Hz, 2H), 1.78 (p, *J* = 7.7 Hz, 2H), 1.50–1.44 (m, 2H), 1.38 – 1.32 (m, 2H); ^{13}C NMR (150 MHz, Chloroform-*d*) δ 162.71, 126.66, 126.41, 126.07, 125.20, 112.86, 99.61, 48.50, 38.83, 33.82, 32.70, 30.81, 27.77, 25.79; HRMS calcd for $C_{14}H_{17}Br_3N_2O$ $[M + H]^+$ 469.8871, found 469.8863.

2,3-dibromo-1,7-bis(4-bromopropyl)-6,7-dihydropyrrolo[2,3-c] azepin-8(1*H*)-one (**9a**)
Yellow oily liquid (1.06 g, yield 56%); ^{1}H NMR (600 MHz, Chloroform-*d*) δ 6.72 (d, *J* = 9.7 Hz, 1H), 6.11 (dt, *J* = 9.6, 6.6 Hz, 1H), 4.50 (t, *J* = 7.2 Hz, 2H), 3.66 (d, *J* = 6.7 Hz, 2H), 3.57 (t, *J* = 7.0 Hz, 2H), 3.42 (dt, *J* = 11.2, 6.4 Hz, 4H), 1.98–1.80 (m, 6H), 1.79–1.69 (m, 2H); ^{13}C NMR (150 MHz, Chloroform-*d*) δ 160.29, 126.89, 126.87, 125.77, 125.41, 112.06, 99.11, 47.52, 46.67, 45.64, 33.41, 32.84, 29.77, 29.60, 28.00; HRMS calcd for $C_{16}H_{20}Br_4N_2O$ $[M + H]^+$ 575.8268, found 577.8265.

2,3-dibromo-1,7-bis(5-bromobutyl)-6,7-dihydropyrrolo[2,3-c] azepin-8(1*H*)-one (**9b**)
Yellow oily liquid (1.03 g, yield 52%); ^{1}H NMR (600 MHz, DMSO-*d6*) δ 6.56 (d, *J* = 9.6 Hz, 1H),

6.15 (q, J = 7.8, 7.4 Hz, 1H), 4.35 (t, J = 7.3 Hz, 2H), 3.62 (d, J = 6.6 Hz, 2H), 3.49 – 3.38 (m, 6H), 1.76 (p, J = 8.7, 7.8 Hz, 4H), 1.66 (q, J = 7.5 Hz, 2H), 1.47 (p, J = 7.3 Hz, 2H), 1.30 (dq, J = 13.8, 7.7 Hz, 4H); ^{13}C NMR (150 MHz, DMSO-*d6*) δ 159.80, 128.08, 127.78, 125.89, 124.61, 111.96, 98.49, 48.06, 47.05, 45.27, 35.46, 35.38, 35.34, 32.46, 32.17, 29.95, 28.47, 25.25, 25.08; HRMS calcd for $C_{18}H_{24}Br_4N_2O$ $[M + H]^+$ 605.8581, found 605.8592.

2,3-dibromo-1,7-bis(6-bromopentyl)-6,7-dihydropyrrolo[2,3-c] azepin-8(1*H*)-one (**9c**)
Yellow oily liquid (1.03 g, yield 50%); ^{1}H NMR (600 MHz, DMSO-*d6*) δ 6.56 (d, J = 9.6 Hz, 1H), 6.14 (q, J = 7.2 Hz, 1H), 4.33 (t, J = 7.4 Hz, 2H), 3.61 (d, J = 6.6 Hz, 2H), 3.47 – 3.38 (m, 6H), 1.73 (q, J = 7.2 Hz, 4H), 1.64 (q, J = 7.5 Hz, 2H), 1.44 (p, J = 7.3 Hz, 2H), 1.34 (q, J = 7.5 Hz, 4H), 1.18 (dq, J = 14.8, 7.3 Hz, 4H); ^{13}C NMR (150 MHz, DMSO-*d6*) δ 159.74, 128.05, 127.78, 125.87, 124.55, 111.89, 98.44, 48.20, 47.26, 45.31, 35.45, 35.34, 32.75, 32.70, 30.69, 29.25, 27.84, 27.65, 25.83, 25.65; HRMS calcd for $C_{20}H_{28}Br_4N_2O$ $[M + H]^+$ 633.8894, found 633.8877.

2. General procedure for Compounds **10a**~**10c**, **11a**~**11c**.

A mixture of compounds **8a**~**8c**, **9a**~**9c** (0.47 mmol), and thiourea (2,36 mmol) in EtOH (10 mL) was heated to reflux for 12 h and monitored with TLC. When the reaction was finished, the solvent was evaporated under reduced pressure, and the resulting residue was purified by chromatography on silica gel to give compounds **10a**~**10c**, **11a**~**11c**.

2-(4-(2,3-dibromo-8-oxo-7,8-dihydropyrrolo[2,3-c]azepin-1(6*H*)-yl)butyl)isothiouronium bromide (**10a**)
White solid (158 mg, yield 67%); ^{1}H NMR (600 MHz, DMSO-*d6*) δ 9.04 (s, 4H), 7.91 (*t*, J = 5.8 Hz, 1H), 6.57 (d, J = 9.9 Hz, 1H), 6.15 (dt, J = 9.9, 6.4 Hz, 1H), 4.45 (t, J = 7.2 Hz, 2H), 3.41 (t, J = 6.1 Hz, 2H), 3.18 (t, J = 7.2 Hz, 2H), 1.85–1.77 (m, 2H), 1.60 (h, J = 7.7, 6.8 Hz, 2H); ^{13}C NMR (150 MHz, DMSO-*d6*) δ 170.19, 161.90, 129.05, 126.83, 125.51, 124.96, 112.28, 98.93, 47.60, 38.08, 30.05, 29.63, 26.31; HRMS calcd for $C_{13}H_{17}Br_2N_4OS$ $[M + H]^+$ 424.9307, found 424.9321.

2-(5-(2,3-dibromo-8-oxo-7,8-dihydropyrrolo[2,3-c]azepin-1(6*H*)-yl)pentyl)isothiouronium bromide (**10b**)
White solid (139 mg, yield 60%); ^{1}H NMR (600 MHz, DMSO-*d6*) δ 8.97 (s, 4H), 7.87 (t, J = 5.8 Hz, 1H), 6.55 (d, J = 9.9 Hz, 1H), 6.12 (dt, J = 9.9, 6.4 Hz, 1H), 4.40 (t, J = 7.4 Hz, 2H), 3.38 (d, J = 12.1 Hz, 2H), 3.11 (t, J = 7.4 Hz, 2H), 1.70 (p, J = 7.5 Hz, 2H), 1.61 (p, J = 7.5 Hz, 2H), 1.33 (p, J = 7.7 Hz, 2H); ^{13}C NMR (150 MHz, DMSO-*d6*) δ 170.31, 161.98, 129.05, 126.84, 125.58, 124.95, 112.32, 98.89, 55.46, 48.01, 38.14, 30.33, 30.26, 28.59, 25.30; HRMS calcd for $C_{14}H_{19}Br_2N_4OS$ $[M + H]^+$ 452.9620, found 452.9613.

2-(6-(2,3-dibromo-8-oxo-7,8-dihydropyrrolo[2,3-c]azepin-1(6*H*)-yl)hexyl)isothiouronium bromide (**10c**)
White solid (163 mg, yield 58%); ^{1}H NMR (600 MHz, DMSO-*d6*) δ 8.98 (s, 4H), 7.84 (t, J = 5.8 Hz, 1H), 6.52 (d, J = 9.9 Hz, 1H), 6.09 (dt, J = 9.9, 6.4 Hz, 1H), 4.36 (t, J = 7.5 Hz, 2H), 3.35 (t, J = 6.1 Hz, 2H), 3.09 (t, J = 7.3 Hz, 2H), 1.65 (p, J = 7.5 Hz, 2H), 1.60–1.52 (m, 2H), 1.35 (p, J = 7.5 Hz, 2H), 1.25 (q, J = 7.6 Hz, 2H); ^{13}C NMR (150 MHz, DMSO-*d6*) δ 170.40, 161.95, 129.02, 126.85, 125.57, 124.90, 112.25, 98.84, 48.14, 38.13, 30.65, 30.52, 28.75, 27.77, 25.80; HRMS calcd for $C_{15}H_{21}Br_2N_4OS$ $[M + H]^+$ 466.9777, found 466.9772.

2,2'-((2,3-dibromo-8-oxo-6,8-dihydropyrrolo[2,3-c]azepine-1,7-diyl)bis(butane-4,1-diyl)) diisothiouronium bromide (**11a**)
Yellow solid (170 mg, yield 67%); ^{1}H NMR (600 MHz, DMSO-*d6*) δ 9.11–8.93 (m, 8H), 6.58 (d, J = 9.6 Hz, 1H), 6.18 (dt, J = 9.7, 6.6 Hz, 1H), 4.35 (t, J = 7.2 Hz, 2H), 3.64 (d, J = 6.6 Hz, 2H), 3.45

(t, *J* = 6.8 Hz, 4H), 3.14 (d, *J* = 7.3 Hz, 2H), 1.76 (p, *J* = 7.5 Hz, 2H), 1.54 (tt, *J* = 9.8, 4.3 Hz, 4H), 1.49–1.43 (m, 2H); ^{13}C NMR (150 MHz, DMSO-*d6*) δ 170.30, 170.24, 159.91,127.21, 128.42, 127.65, 125.84, 124.78, 112.27, 98.60, 49.11, 47.78, 46.67, 45.41, 30.27, 30.09, 29.65, 28.22, 26.43, 26.26; HRMS calcd for $C_{18}H_{28}Br_2N_6OS_2$ [M + H]$^+$ 570.3899, found 570.3893.

2,2'-((2,3-dibromo-8-oxo-6,8-dihydropyrrolo[2,3-c]azepine-1,7-diyl)bis(pentane-5,1-diyl)) diisothiouronium bromide (**11b**)

Yellow solid (149 mg, yield 60%); ^{1}H NMR (600 MHz, DMSO-*d6*) δ 8.96 (d, *J* = 42.4 Hz, 8H), 6.58 (d, *J* = 9.6 Hz, 1H), 6.18 (dt, *J* = 9.6, 6.6 Hz, 1H), 4.33 (t, *J* = 7.4 Hz, 2H), 3.64 (d, *J* = 6.6 Hz, 2H), 3.41 (t, *J* = 7.1 Hz, 2H), 3.13–3.05 (m, 5H), 1.68 (p, *J* = 7.5 Hz, 2H), 1.56 (q, *J* = 7.5 Hz, 4H), 1.47 (q, *J* = 7.4 Hz, 2H), 1.32–1.24 (m, 4H); ^{13}C NMR (150 MHz, DMSO-*d6*) δ 170.38, 170.33, 159.78, 128.35, 127.66, 125.73, 124.66, 98.44, 60.25, 48.08, 47.08, 45.39, 30.49, 30.35, 30.21, 28.83, 28.53, 28.40, 25.37, 25.27, 21.24, 14.56; HRMS calcd for $C_{20}H_{32}Br_2N_6OS_2$ [M + H]$^+$ 598.0435, found 598.0431.

2,2'-((2,3-dibromo-8-oxo-6,8-dihydropyrrolo[2,3-c]azepine-1,7-diyl)bis(hexane-6,1-diyl)) diisothiouronium bromide (**11c**)

Yellow solid (142 mg, yield 57%); ^{1}H NMR (600 MHz, DMSO-*d6*) δ 8.95 (s, 8H), 6.57 (d, *J* = 9.6 Hz, 1H), 6.17 (dt, *J* = 9.6, 6.6 Hz, 1H), 4.32 (t, *J* = 7.4 Hz, 2H), 3.63 (d, *J* = 6.6 Hz, 2H), 3.40 (t, *J* = 7.2 Hz, 2H), 3.09 (td, *J* = 7.3, 1.4 Hz, 4H), 1.65 (p, *J* = 7.6 Hz, 2H), 1.53 (p, *J* = 7.8 Hz, 4H), 1.43 (p, *J* = 7.3 Hz, 2H), 1.36–1.30 (m, 4H), 1.24–1.18 (m, 4H); ^{13}C NMR (150 MHz, DMSO-*d6*) δ 170.88, 170.38, 159.74, 128.32, 127.70, 125.72, 124.58, 111.96, 98.38, 60.26, 48.18, 47.25, 45.37, 30.58, 30.53, 30.49, 29.26, 28.70, 28.66, 27.91, 27.77, 25.93, 25.78, 21.24, 14.55; HRMS calcd for $C_{22}H_{36}Br_2N_6OS_2$ [M + H]$^+$ 626.4979, found 626.4983.

3. Tabel S1: The high-throughput-screen result of **aldisine** derivatives.

Compounds	Luciferase intensity (%) [1]	
	concentration of compound (5μM)	concentration of compound (20μM)
**10a**	76.46	−9.28
**10b**	61.68	−6.02
**10c**	48.79	−8.85
**11a**	77.08	−9.04
**11b**	98.13	−7.35
**11c**	42.08	−7.93

[1]The luciferase intensity of the compounds were analyzed by a STAT3 transcriptional activity-based high-throughput luciferase reporter screen system. The lower the luciferase intensity, the higher the STAT3 inhibitory activity.

4.

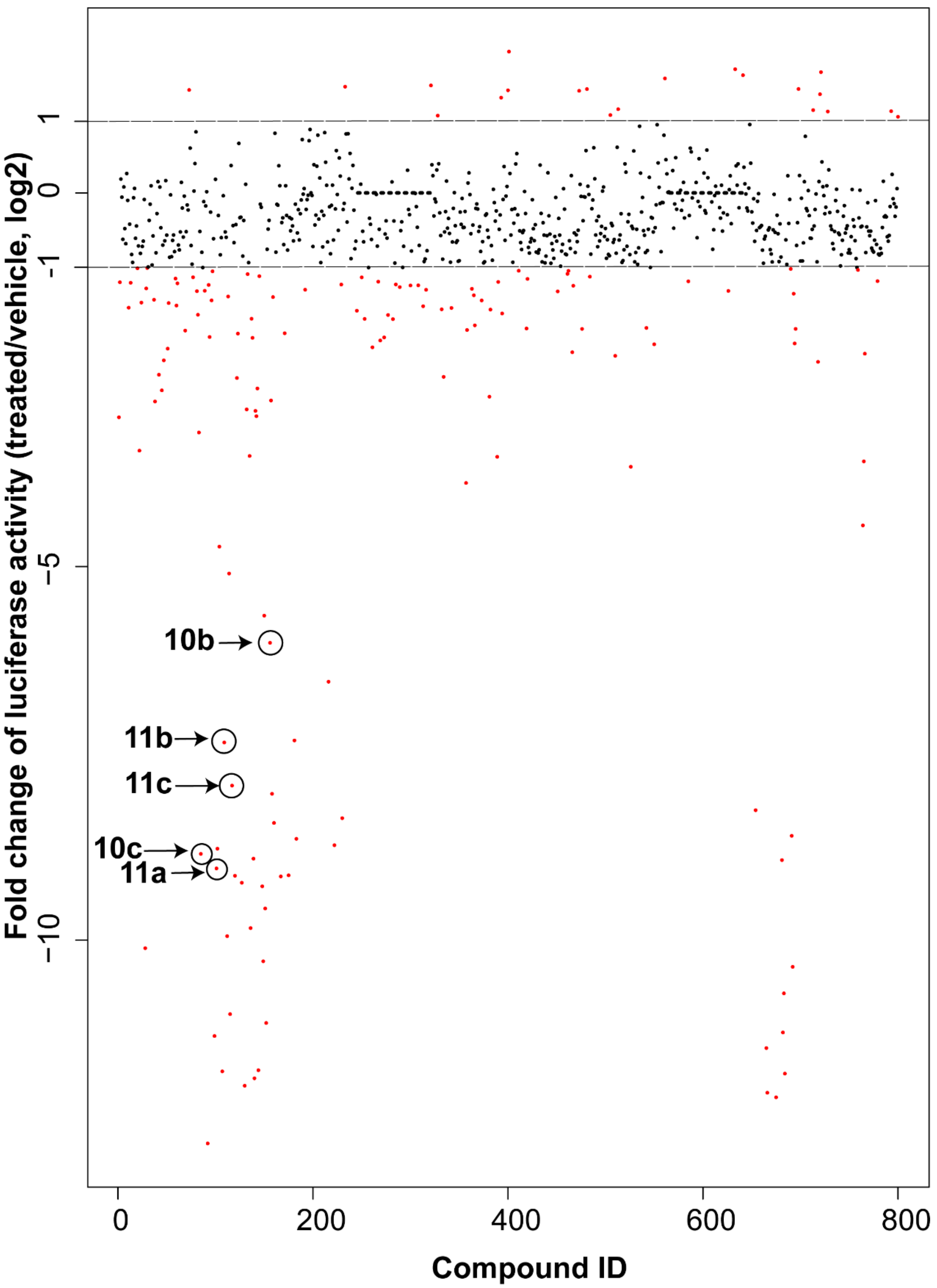

Figure S1: A graph of the high-throughput screen of compounds.

5. The data of ^{1}H and ^{13}C NMR of all the compounds.

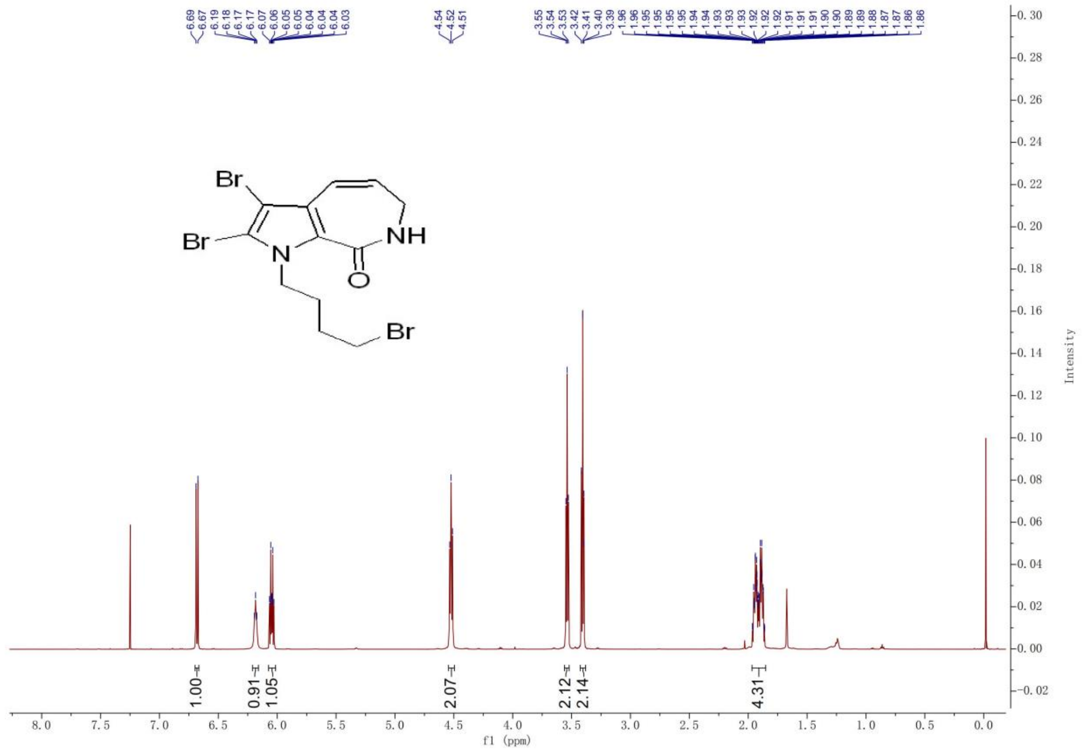

^{1}H NMR Spectrum of **8a** in $CDCl_3$

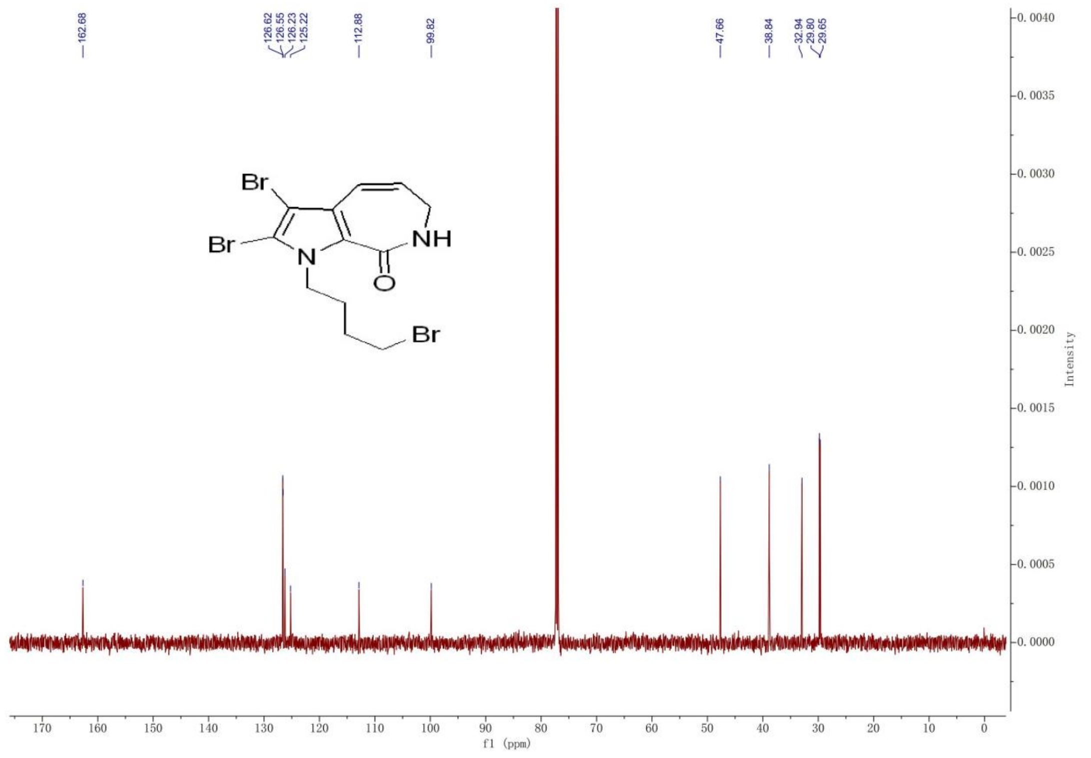

^{13}C NMR Spectrum of **8a** in $CDCl_3$

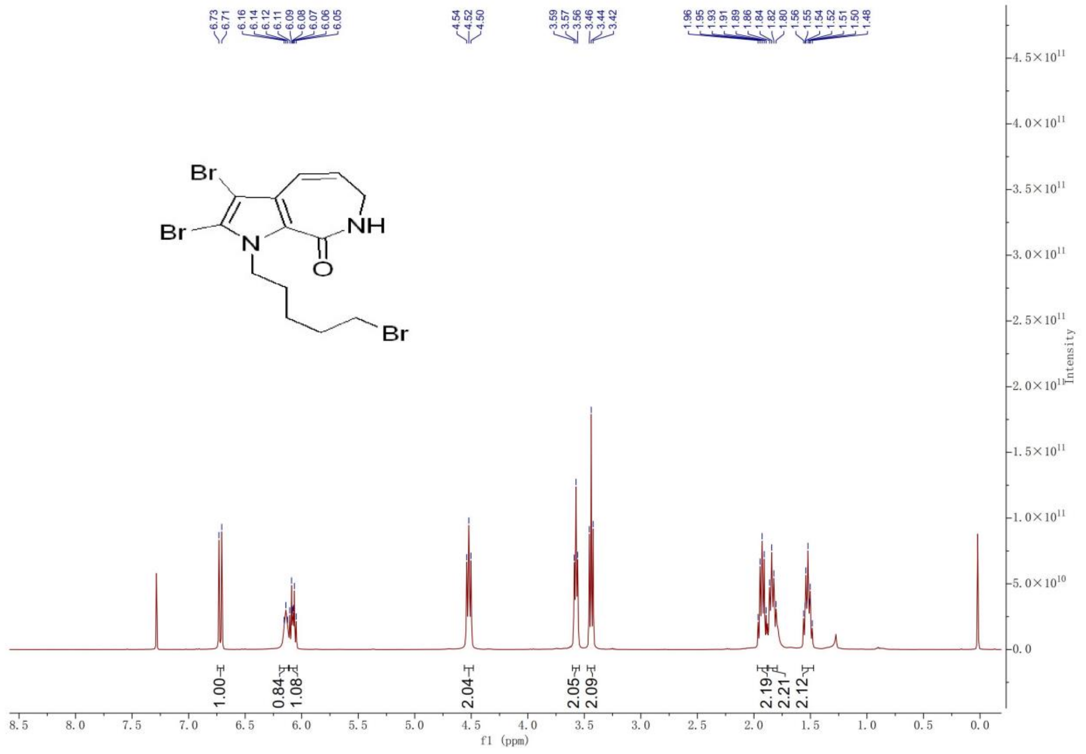

^{1}H NMR Spectrum of **8b** in $CDCl_3$

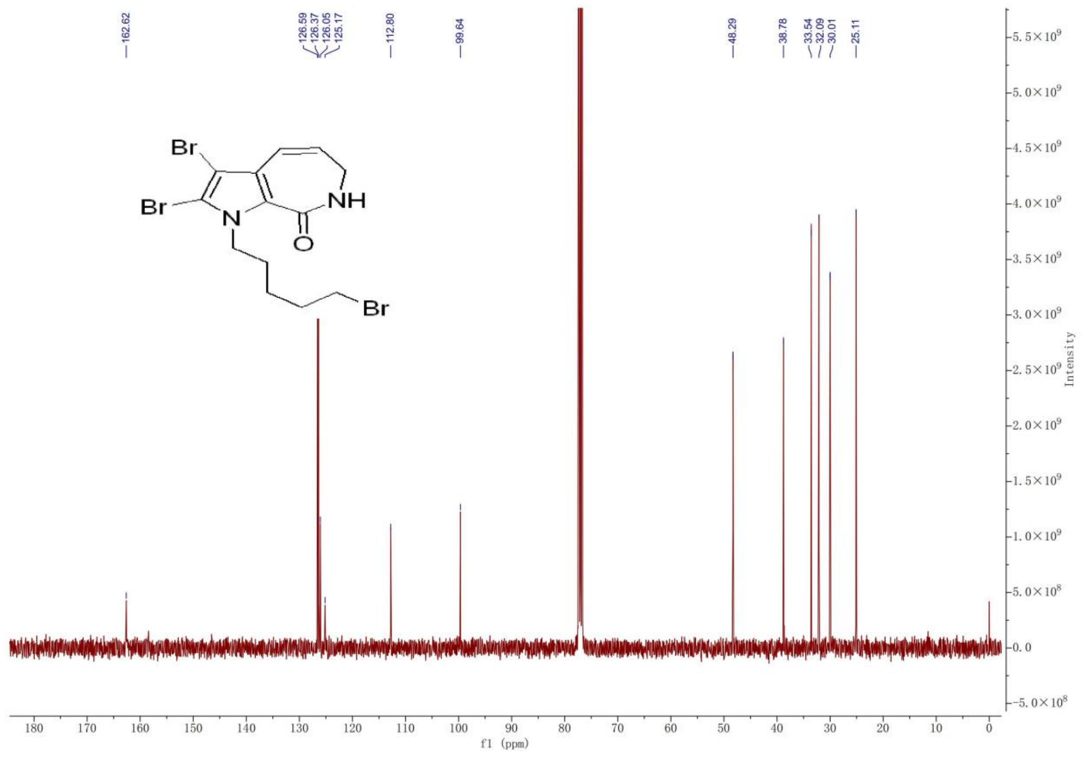

^{13}C NMR Spectrum of **8b** in $CDCl_3$

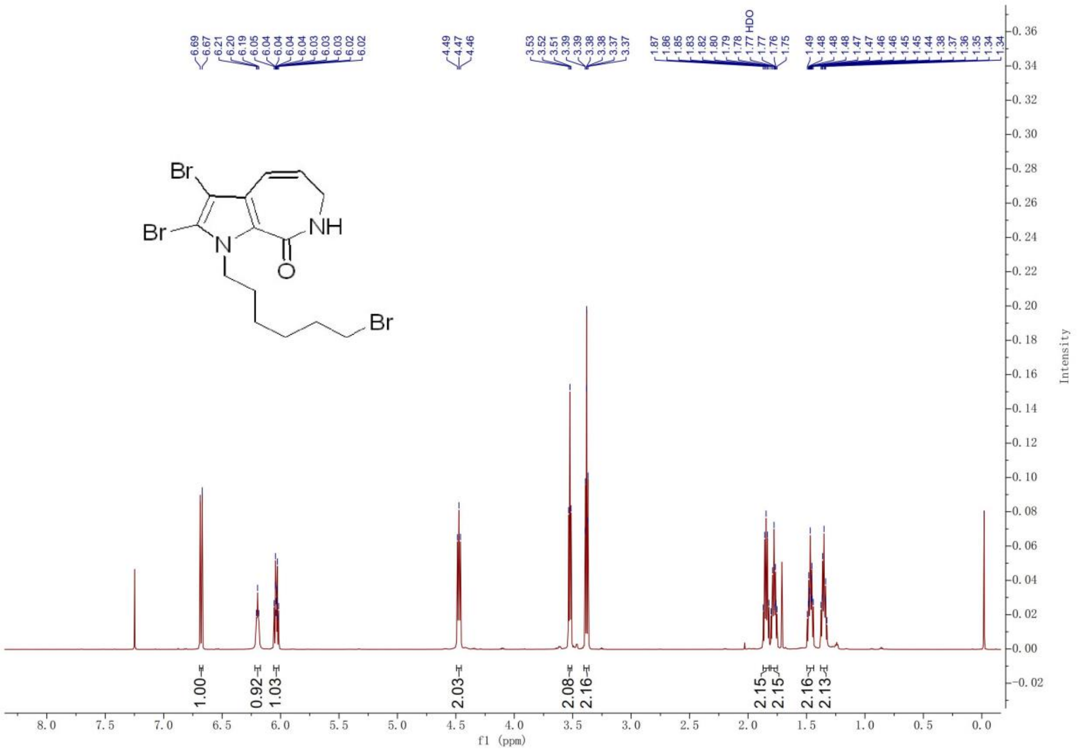

^{1}H NMR Spectrum of **8c** in $CDCl_3$

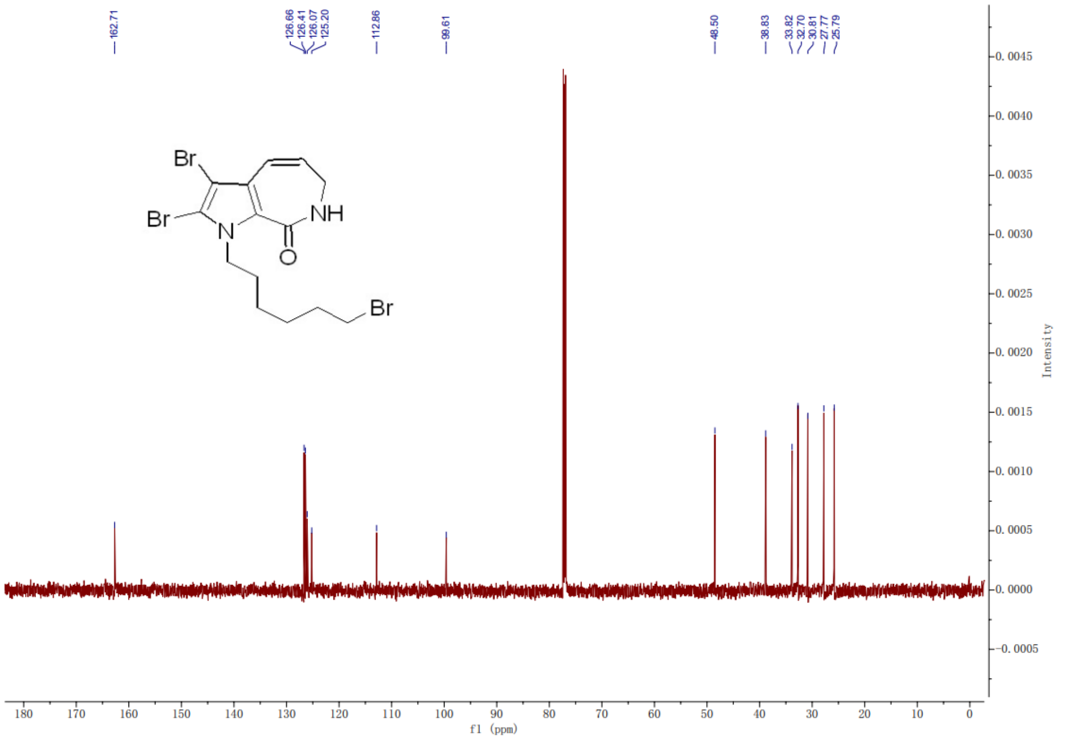

^{13}C NMR Spectrum of **8c** in $CDCl_3$

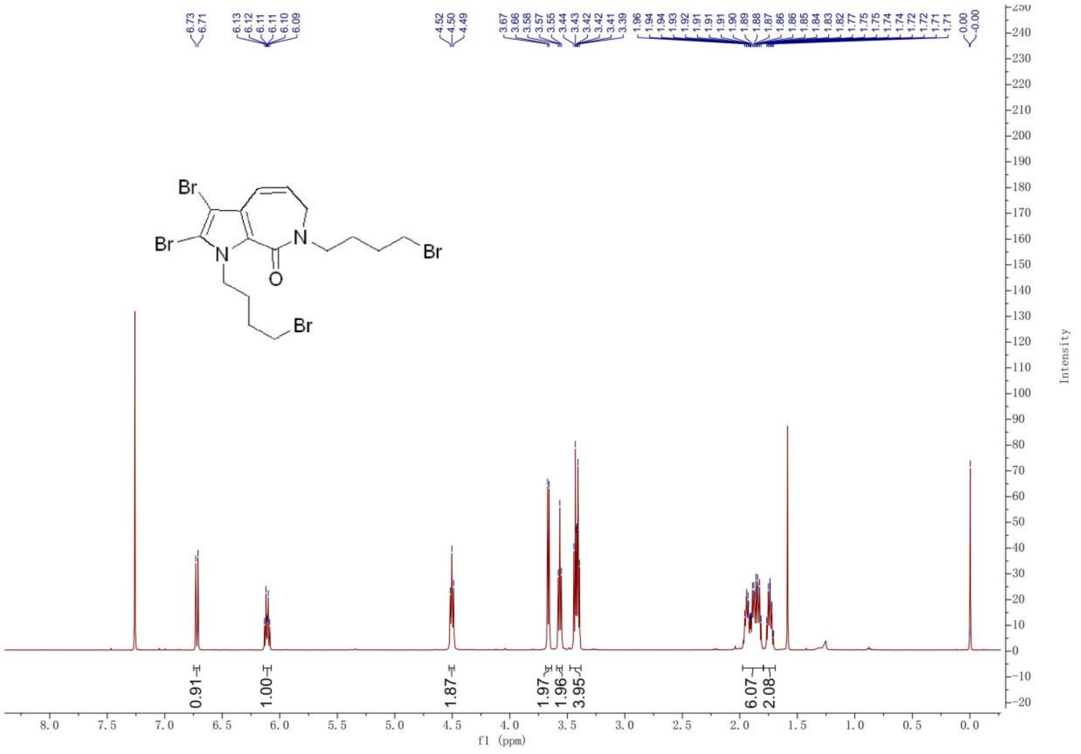

^{1}H NMR Spectrum of **9a** in $CDCl_3$

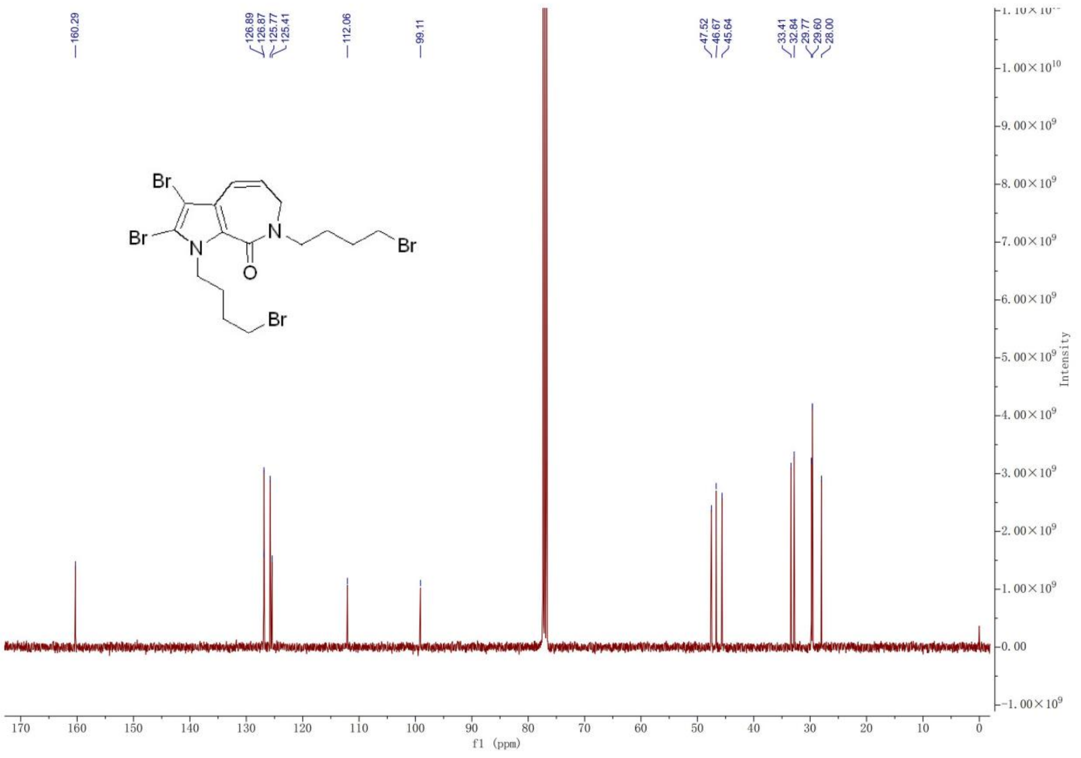

^{13}C NMR Spectrum of **9a** in $CDCl_3$

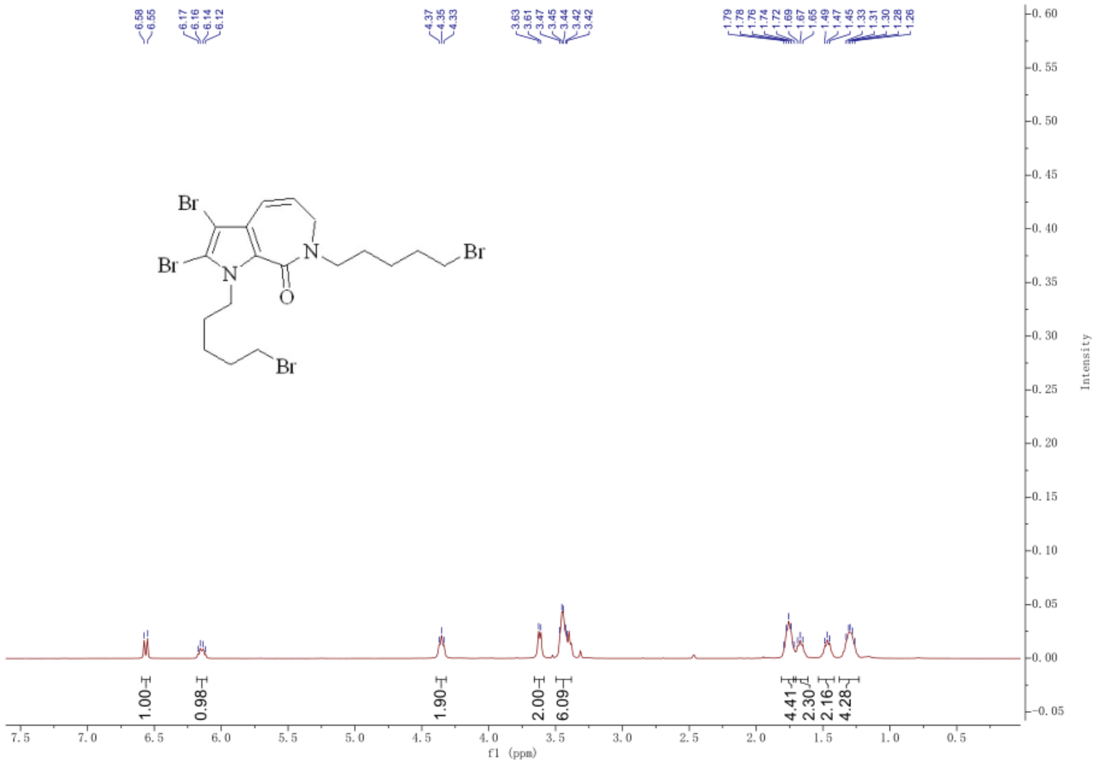

^{1}H NMR Spectrum of **9b** in DMSO-d_6

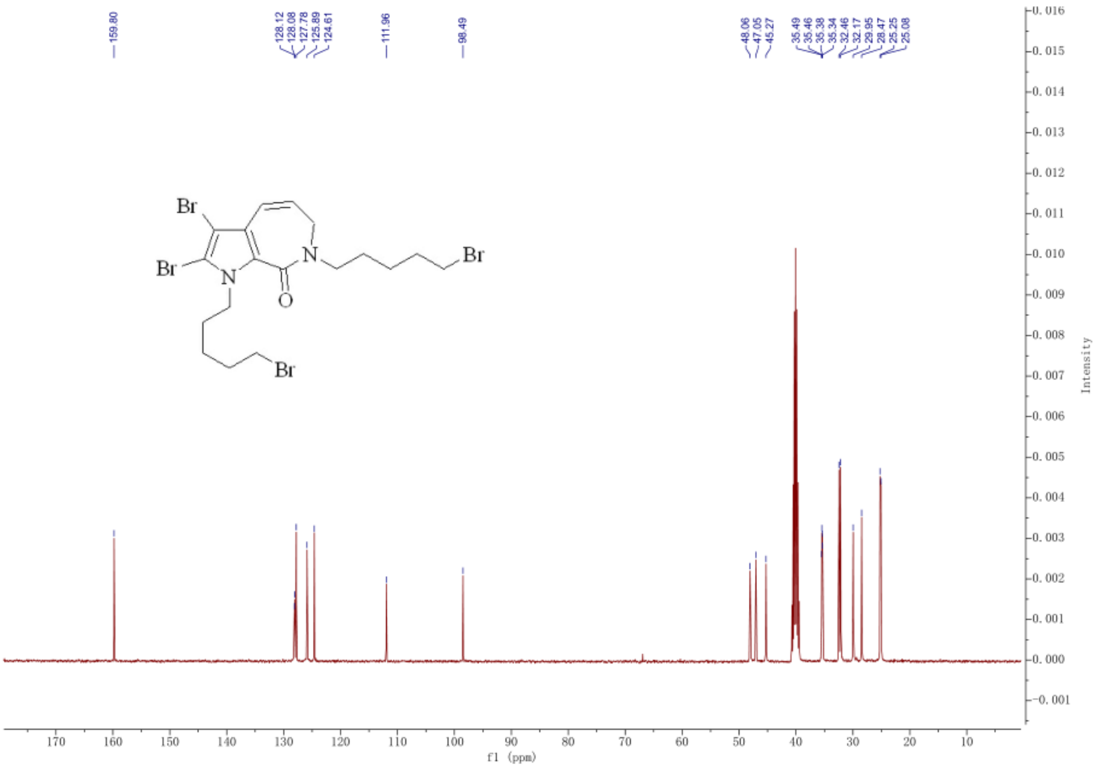

^{13}C NMR Spectrum of **9b** in DMSO-d_6

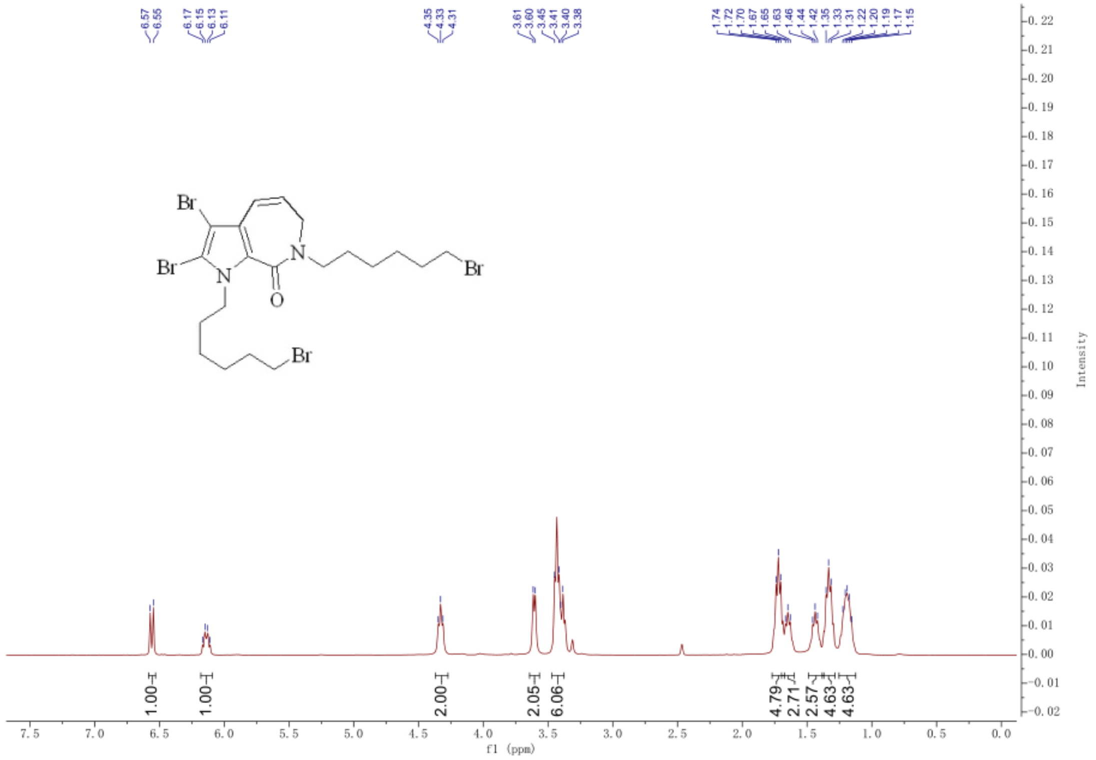

^{1}H NMR Spectrum of **9c** in DMSO-d_6

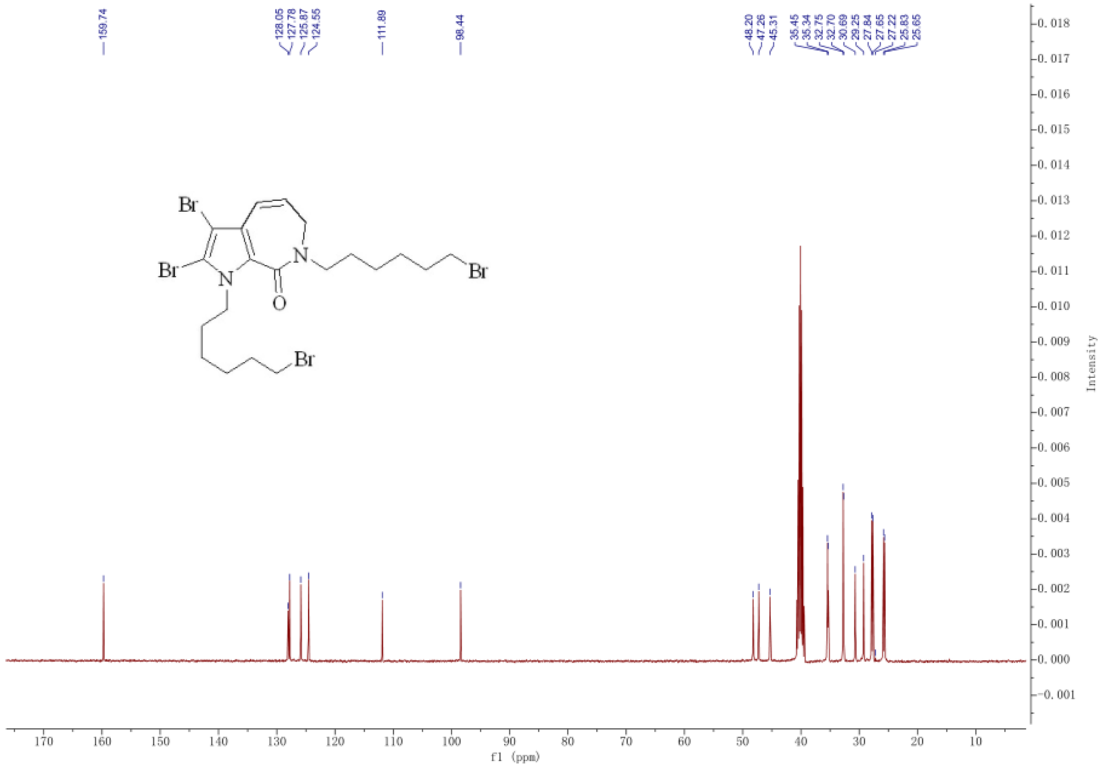

^{13}C NMR Spectrum of **9c** in DMSO-d_6

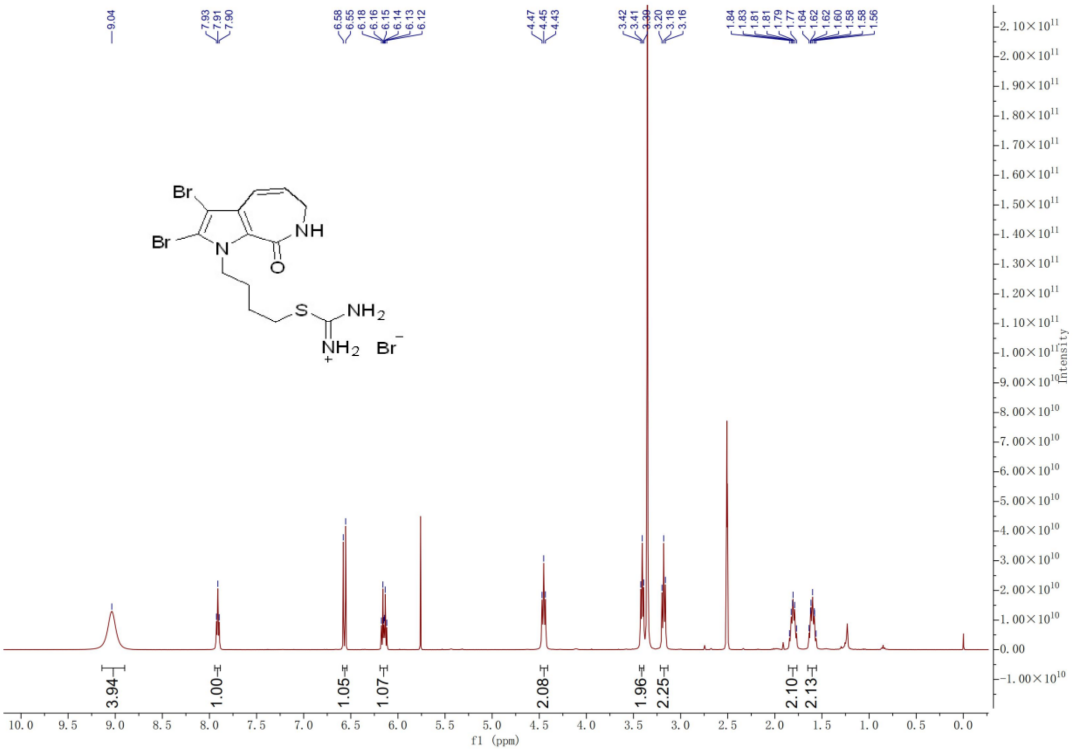

^{1}H NMR Spectrum of **10a** in DMSO-d_6

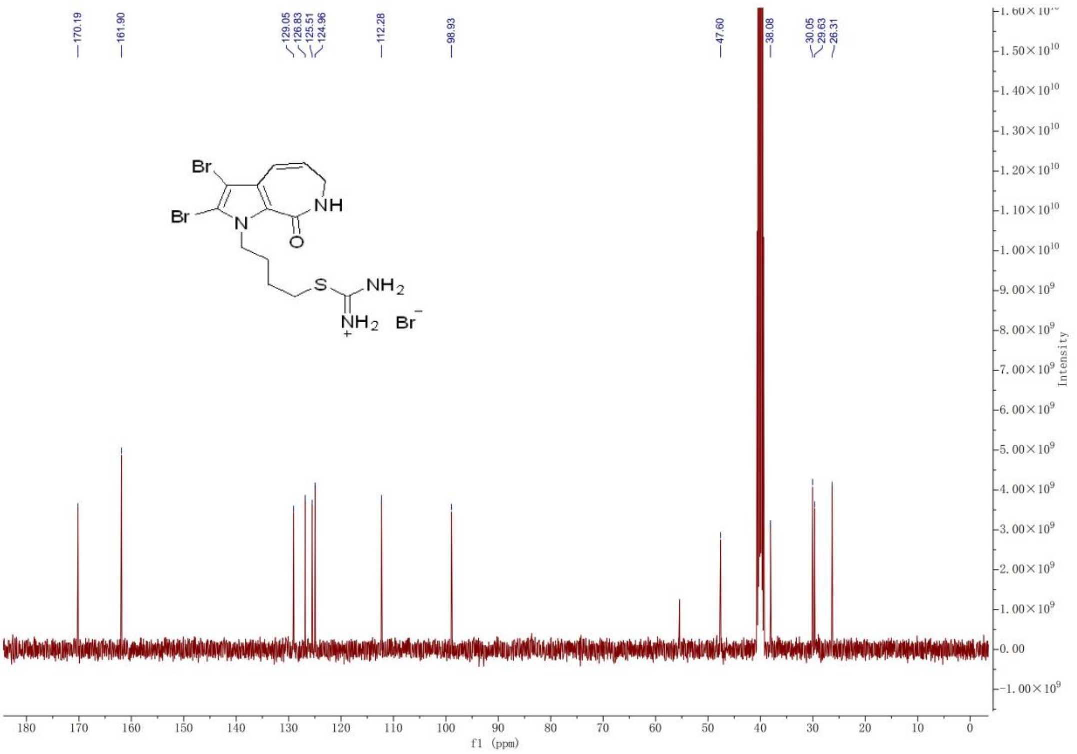

^{13}C NMR Spectrum of **10a** in DMSO-d_6

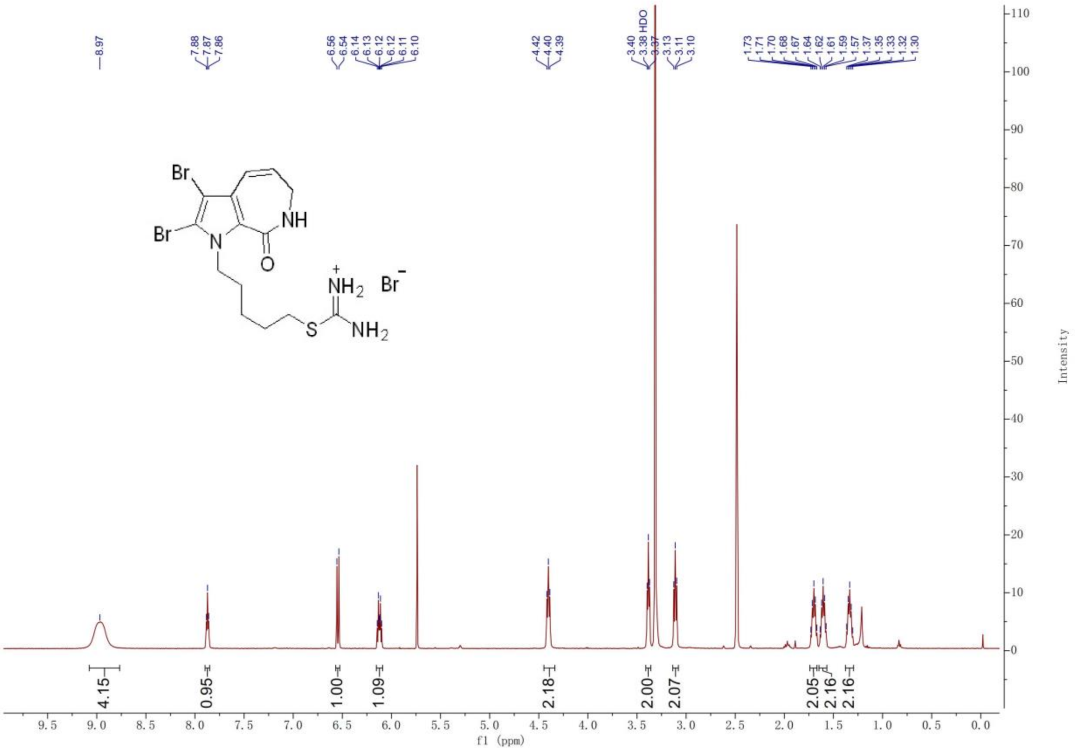

^{1}H NMR Spectrum of **10b** in DMSO-d_6

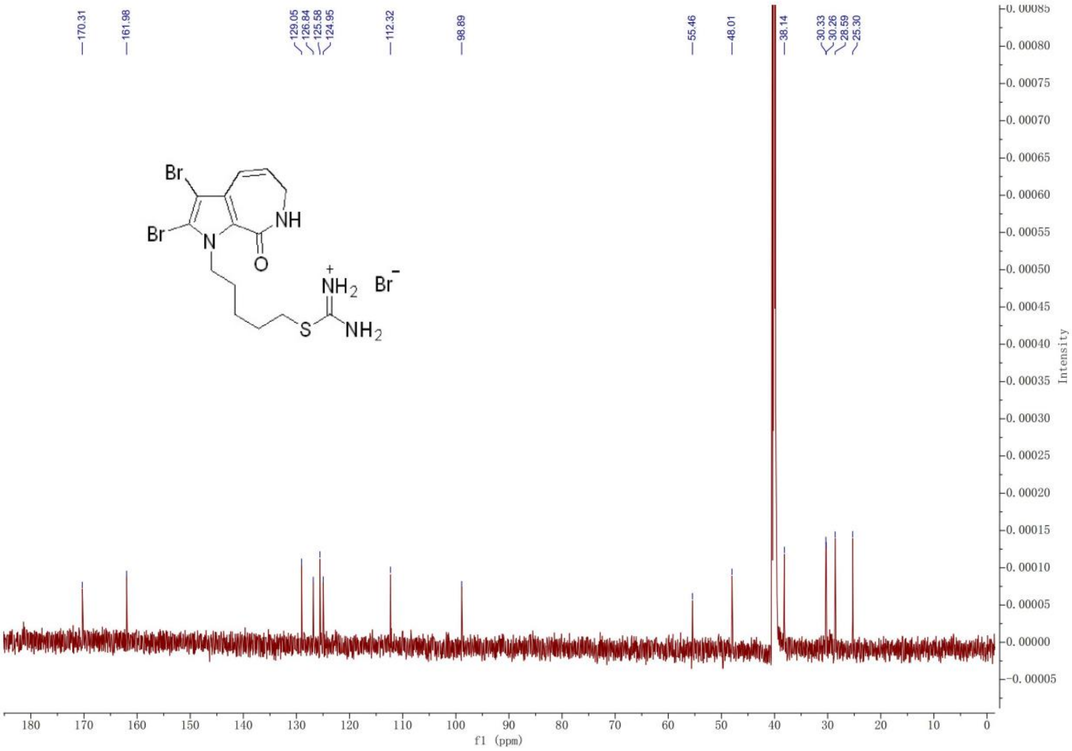

^{13}C NMR Spectrum of **10b** in DMSO-d_6

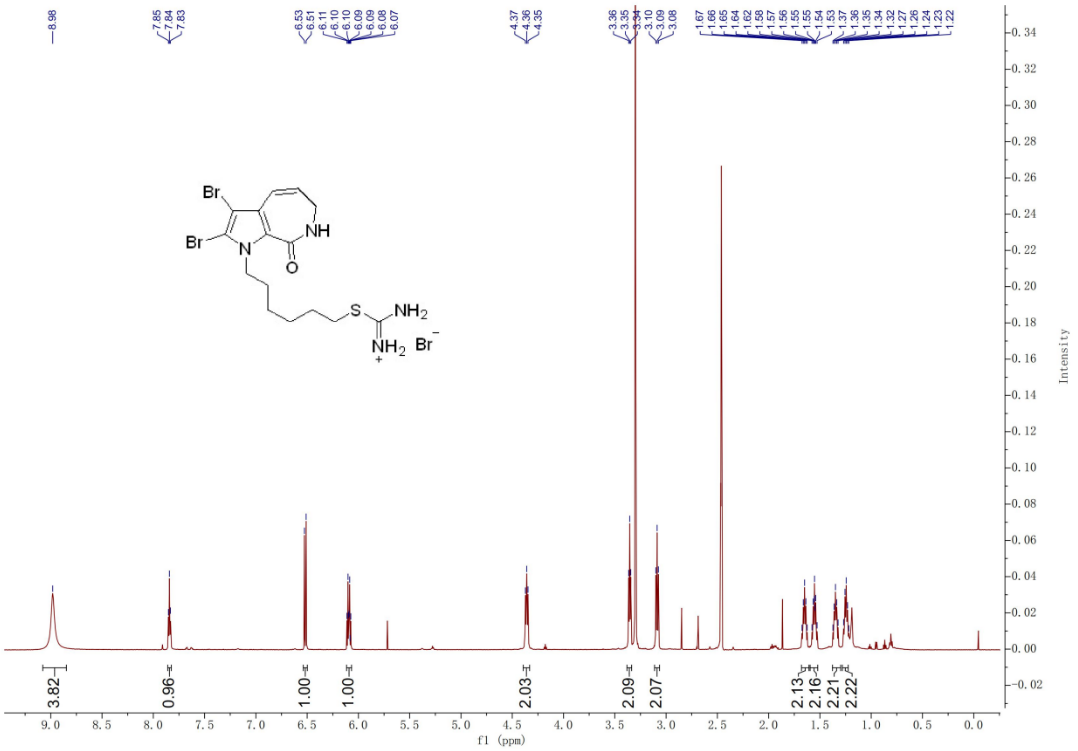

^{1}H NMR Spectrum of **10c** in DMSO-d_6

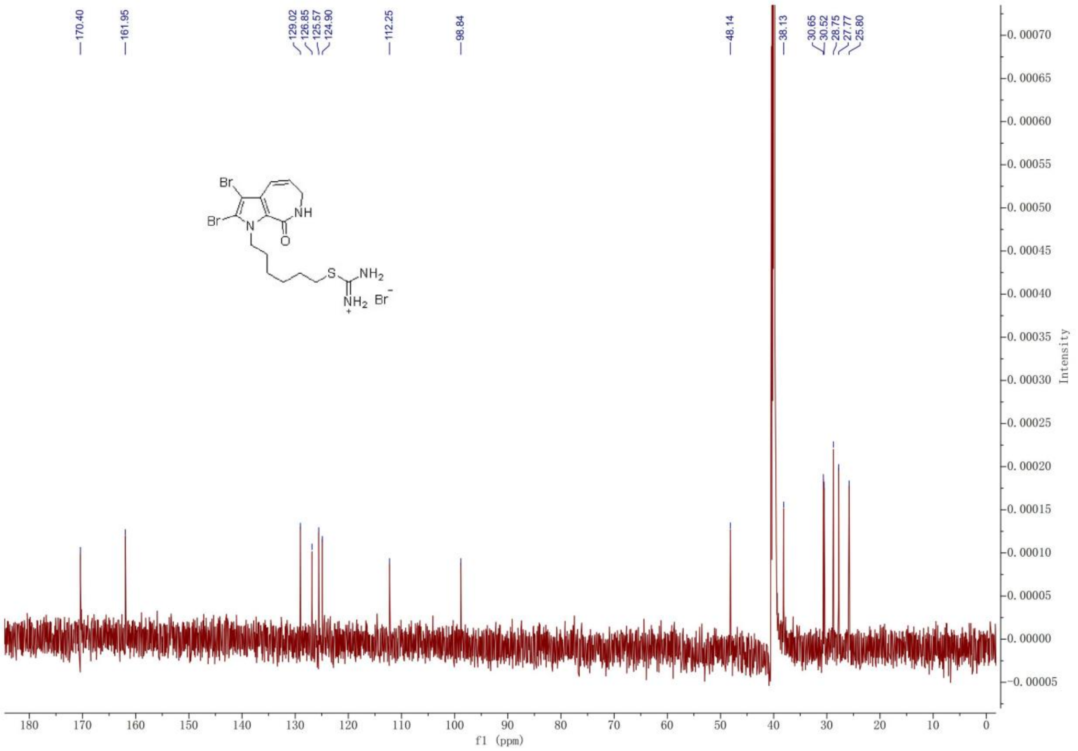

^{13}C NMR Spectrum of **10c** in DMSO-d_6

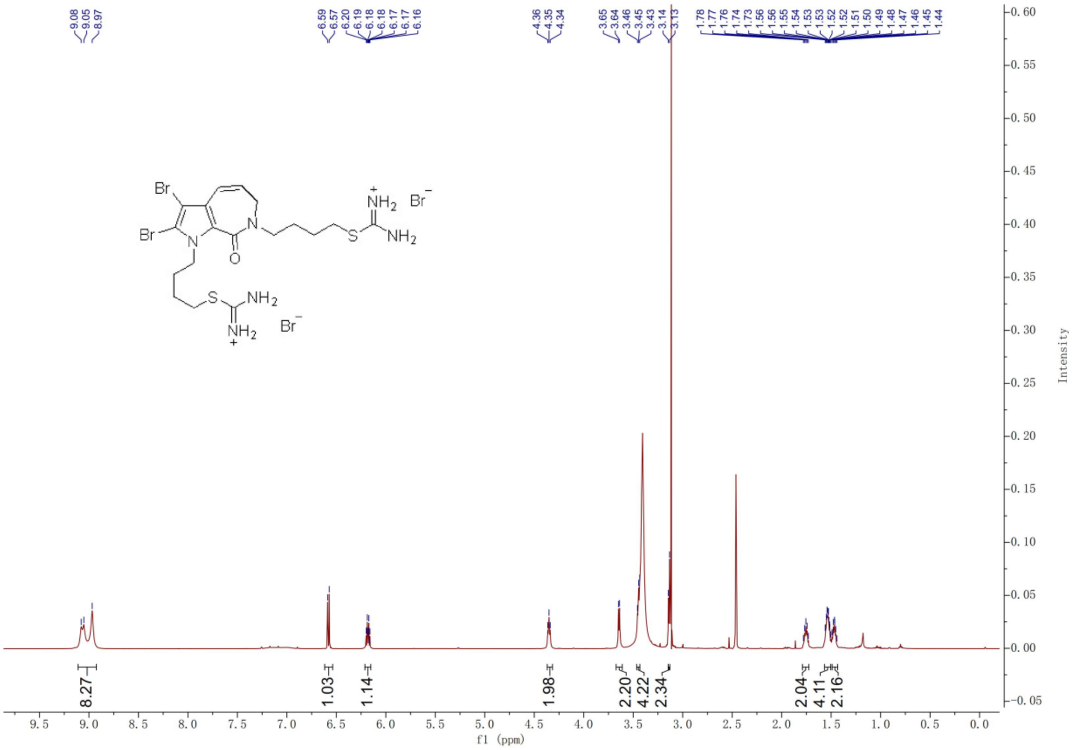

^{1}H NMR Spectrum of **11a** in DMSO-d_6

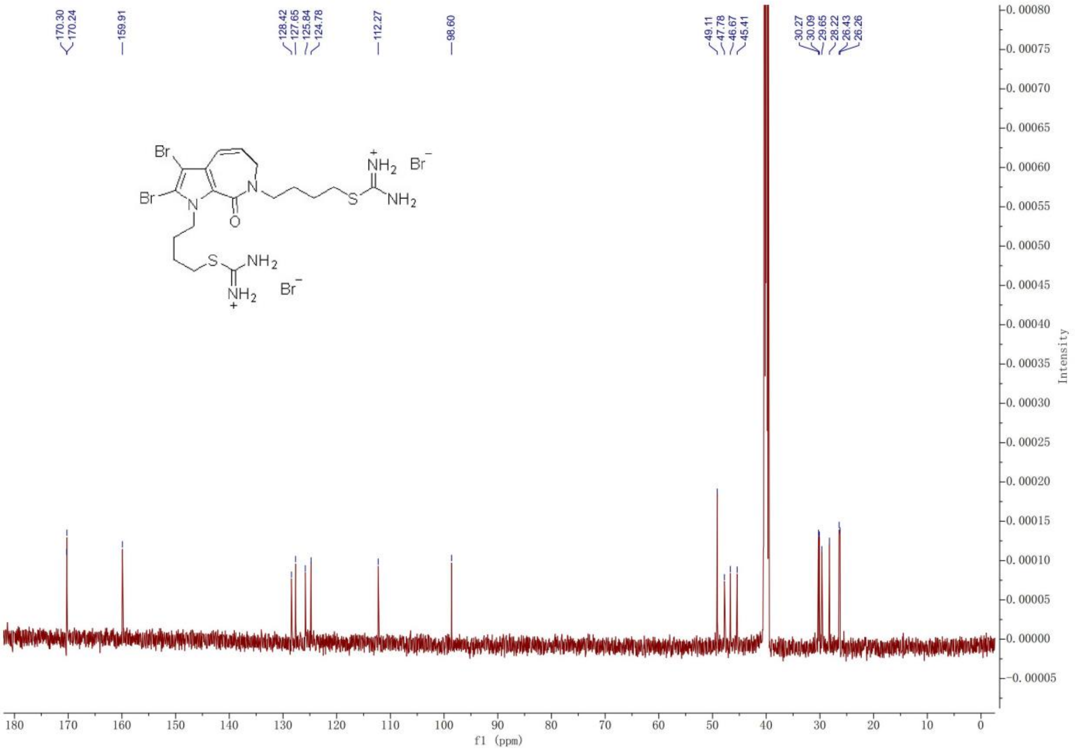

^{13}C NMR Spectrum of **11a** in DMSO-d_6

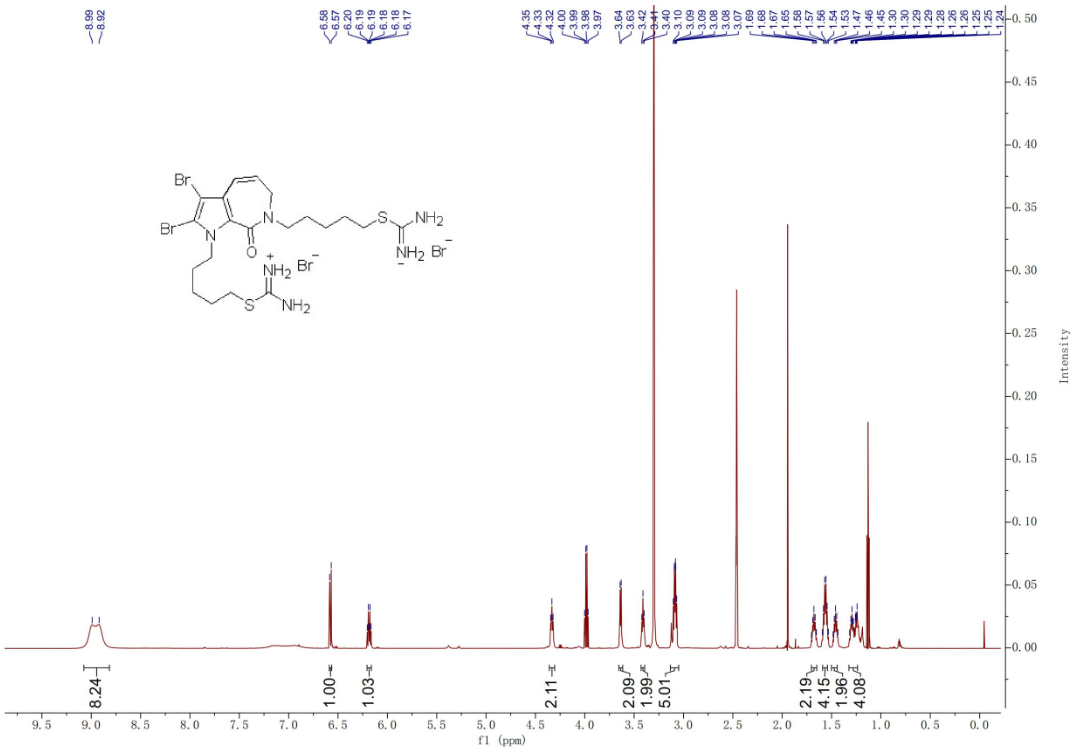

^{1}H NMR Spectrum of **11b** in DMSO-d_6

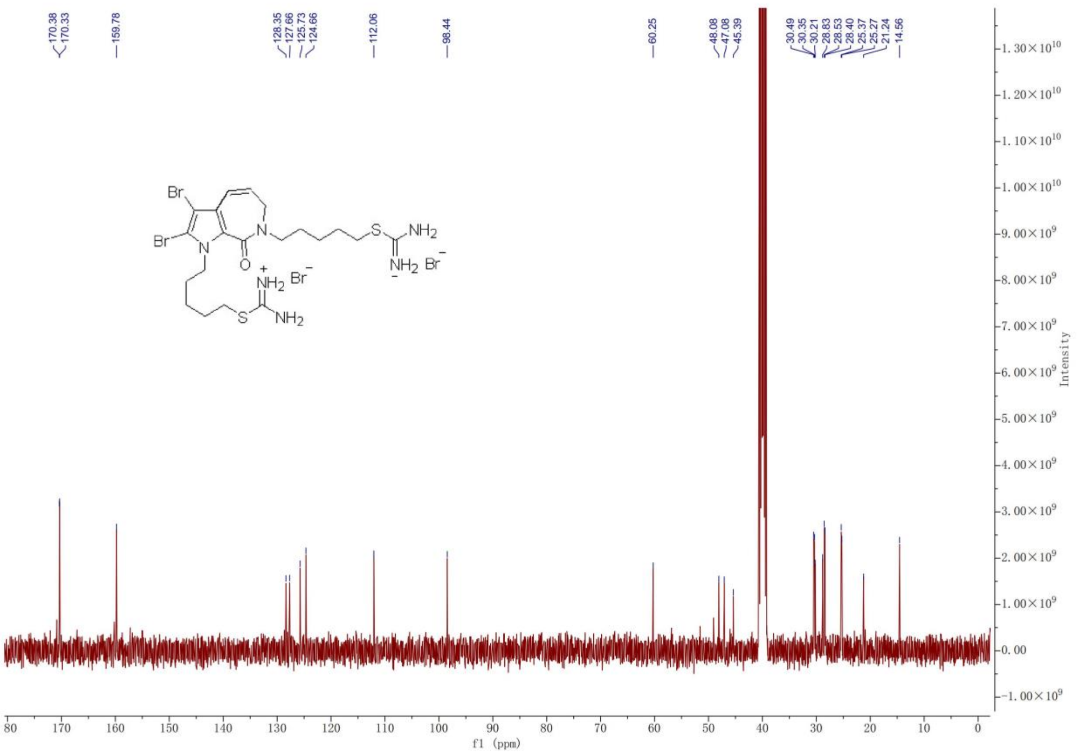

^{13}C NMR Spectrum of **11b** in DMSO-d_6

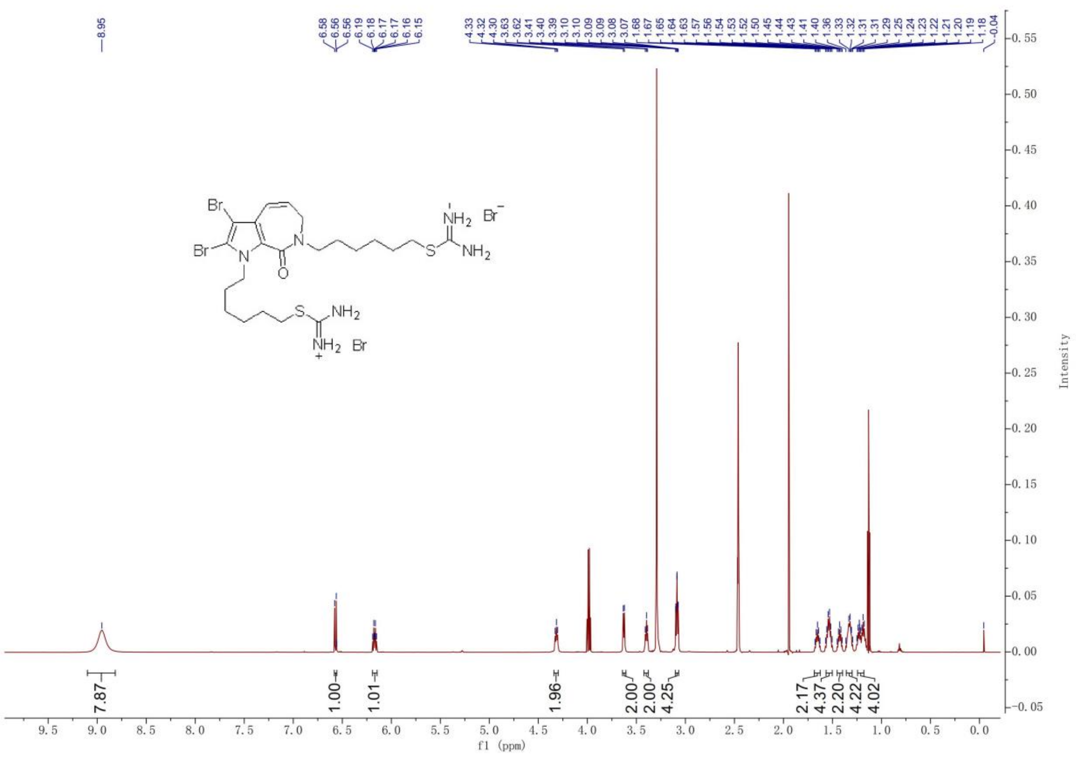

^{1}H NMR Spectrum of **11c** in DMSO-d_6

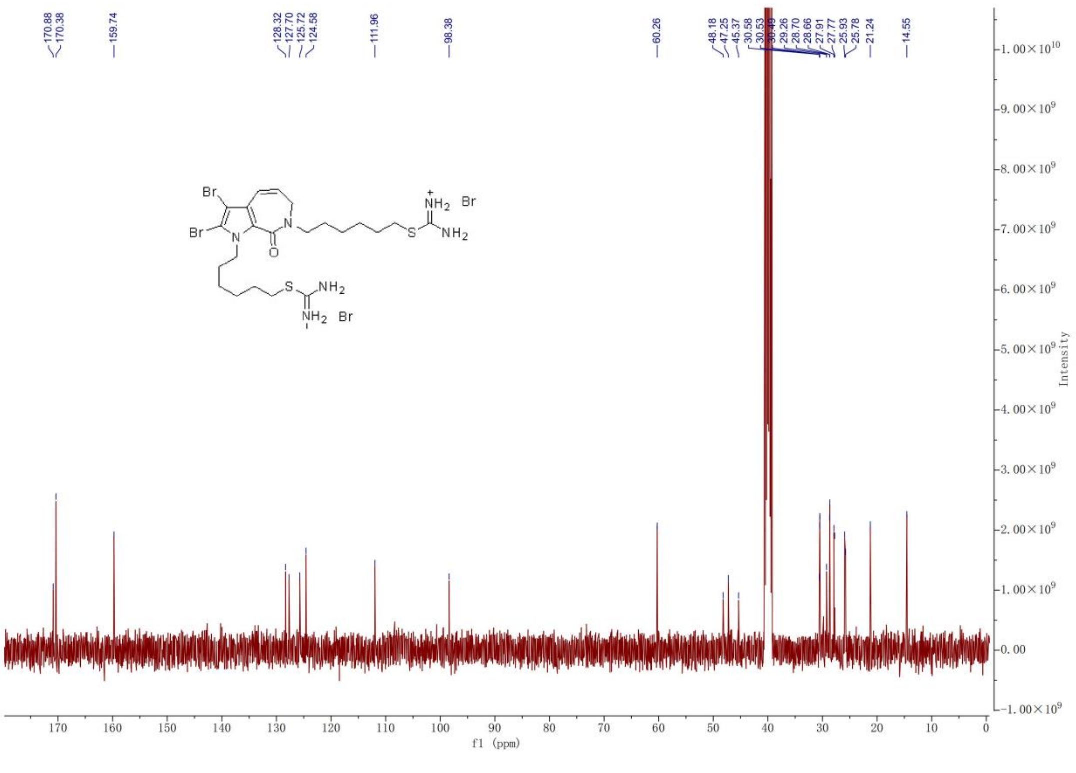

^{13}C NMR Spectrum of **11c** in DMSO-d_6
